# Management of insecticide resistance in the major Aedes vectors of arboviruses: Advances and challenges

**DOI:** 10.1371/journal.pntd.0007615

**Published:** 2019-10-10

**Authors:** Isabelle Dusfour, John Vontas, Jean-Philippe David, David Weetman, Dina M. Fonseca, Vincent Corbel, Kamaraju Raghavendra, Mamadou B. Coulibaly, Ademir J. Martins, Shinji Kasai, Fabrice Chandre

**Affiliations:** 1 Laboratoire d’Entomologie Médicale, Institut Pasteur de la Guyane, Cayenne, French Guiana, France; 2 Institute of Molecular Biology and Biotechnology, Foundation for Research and Technology-Hellas (FORTH), Heraklion, Crete, Greece; 3 Pesticide Science Laboratory, Agricultural University of Athens, Athens, Greece; 4 Laboratoire d’Ecologie Alpine (LECA), Centre National de la Recherche Scientifique (CNRS), Université Grenoble-Alpes, Grenoble, France; 5 Department of Vector Biology, Liverpool School of Tropical Medicine (LSTM), Liverpool, United Kingdom; 6 Center for Vector Biology, Rutgers University (RU), New Brunswick, New Jersey, United States of America; 7 Maladies Infectieuses et Vecteurs, Ecologie, Génétique, Evolution et Contrôle (MIVEGEC), Institut de Recherche pour le Développement, IRD, CNRS, University of Montpellier, Montpellier, France; 8 Department of Health Research, National Institute of Malaria Research, Dwarka, Delhi, India; 9 Malaria Research and Training Center (MRTC), University of Sciences, Techniques and Technologies of Bamako, Bamako, Mali; 10 Laboratório de Fisiologia e Controle de Artrópodes Vetores, Instituto Oswaldo Cruz (FIOCRUZ), Rio de Janeiro, Brazil; 11 Laboratory of Pesticide Science, Department of Medical Entomology, National Institute of Infectious Diseases, Tokyo, Japan; Vienna, AUSTRIA

## Abstract

**Background:**

The landscape of mosquito-borne disease risk has changed dramatically in recent decades, due to the emergence and reemergence of urban transmission cycles driven by invasive *Aedes aegypti* and *Ae*. *albopictus*. Insecticide resistance is already widespread in the yellow fever mosquito, *Ae*. *Aegypti*; is emerging in the Asian tiger mosquito *Ae*. *Albopictus*; and is now threatening the global fight against human arboviral diseases such as dengue, yellow fever, chikungunya, and Zika. Because the panel of insecticides available for public health is limited, it is of primary importance to preserve the efficacy of existing and upcoming active ingredients. Timely implementation of insecticide resistance management (IRM) is crucial to maintain the arsenal of effective public health insecticides and sustain arbovirus vector control.

**Methodology and principal findings:**

This Review is one of a series being generated by the Worldwide Insecticide resistance Network (WIN) and aims at defining the principles and concepts underlying IRM, identifying the main factors affecting the evolution of resistance, and evaluating the value of existing tools for resistance monitoring. Based on the lessons taken from resistance strategies used for other vector species and agricultural pests, we propose a framework for the implementation of IRM strategies for *Aedes* mosquito vectors.

**Conclusions and significance:**

Although IRM should be a fixture of all vector control programs, it is currently often absent from the strategic plans to control mosquito-borne diseases, especially arboviruses. Experiences from other public health disease vectors and agricultural pests underscore the need for urgent action in implementing IRM for invasive *Aedes* mosquitoes. Based on a plan developed for malaria vectors, here we propose some key activities to establish a global plan for IRM in *Aedes* spp.

## Author summary

Arthropod-borne viruses transmitted by *Aedes aegypti* and *Ae*. *albopictus* represent a major public health concern at a global scale. The insecticidal treatments exerted on both species have selected for various resistance mechanisms within wild populations. Although the impact of insecticide resistance on the efficacy of vector control operations remains broadly unknown, it is of primary importance to implement strategies for preserving the efficacy of treatments and reducing the pathogen transmission during epidemics. For this purpose, there are urgent needs for new tools for vector control and insecticide resistance monitoring to improve the management of insecticide resistance in *Aedes* species.

## Introduction

The landscape of mosquito-borne disease risk has changed dramatically in recent decades, due to the emergence and reemergence of urban transmission cycles driven by invasive *Aedes aegypti*, the yellow fever mosquito, and *Ae*. *albopictus*, the Asian tiger mosquito [1]. Prevention of *Aedes*-driven viral diseases, such as Zika, dengue, chikungunya, and even yellow fever, for which the vaccine is effective but supply limited, depends primarily on *Aedes* control or the interruption of human–*Aedes* contact [2]. Conventional control strategies focus on the reduction of larval habitats and density using mechanical, biological, or chemical methods and the reduction of adult populations using mostly chemical insecticides. However, insecticide resistance is already widespread in *Ae*. *aegypti* and is increasing in *Ae*. *albopictus*, threatening the global fight against human arboviral diseases [3–5].

The situation is critical: (1) the panel of insecticides available for public health is very limited [6], and (2) insecticide-based strategies remain the most readily implemented at a global scale and are essential for emergency outbreak situations. Therefore, insecticide resistance management (IRM) is crucial. The primary objectives of IRM are to prevent the emergence of resistance in susceptible populations, to slow its evolution, or to reverse it to a level compatible with an efficient use of insecticides for vector control, while minimizing negative effects on the environment. IRM starts by careful monitoring of insecticide resistance in space and time while evaluating its impact on vector control activities. If insecticide resistance is detected before it has an operational impact, various IRM strategies can be applied depending on the biology of the targeted species, the level of resistance, the nature of resistance mechanisms, and pesticide pressures together with logistical, administrative, and political constraints.

This Review first aims to (i) identify the main factors affecting the evolution of resistance, (ii) define the principles and concepts underlying IRM, and (iii) evaluate the value of existing monitoring tools and the implementation of resistance monitoring in control programs. Then, the different IRM strategies applicable for *Aedes* mosquito vectors are reviewed with examination of available data and lessons from IRM strategies used for other vector species and agricultural pests. Finally, a roadmap toward a global plan for IRM in *Aedes* spp. is proposed.

## IRM, a need for sustaining the control of arbovirus mosquito vectors

Insecticide resistance is defined as an inherited ability of a population to survive an insecticide dose that would normally have proven lethal to individuals of a susceptible population of the same species administrated under the same conditions [7]. Despite concerns of significant insecticide resistance, very few studies have addressed its impact on the control of *Aedes* mosquitoes in the field. All studies referred to the consequences of resistance on entomological outcomes but not on the virus’s transmission or disease incidence. In the larval stages, insecticide resistance is associated with a reduction of treatment efficacy or residual efficacy. For example, in the Caribbean [8], a susceptible population of *Ae*. *aegypti* was fully controlled by a slow-release temephos formulation for up to 8 weeks, but the duration of efficacy did not exceed 3 weeks in populations exhibiting moderate resistance (5-fold resistance ratio), while for highly resistant populations (15-fold resistance ratio), mortality rates decreased below 50% after only 1 week. Similarly, in Brazil, populations exhibiting resistance had reduced mortality in temephos-treated containers compared with a susceptible reference strain [9]. In the case of adulticides, small-scale field trials conducted in the island of Martinique demonstrated that pyrethroid resistance of *Ae*. *aegypti* reduced the efficacy of space spraying [10, 11]. Indeed, no reduction in either larval or adult *Ae*. *aegypti* densities was reported in 9 localities after 3 rounds of thermal fogging with pyrethroids. Additionally, sentinel cages harboring local *Ae*. *aegypti* did not show mortality after treatment, whereas laboratory-susceptible mosquitoes were readily killed. Follow-up genetic studies revealed the presence of multiple resistance mechanisms, including target-site mutation and metabolic enzymes [12, 13]. Similar results were obtained in French Guiana, where cage bioassays showed strongly reduced efficacy of deltamethrin against adults [14]. In that same study, fenitrothion, an organophosphate insecticide, appeared to retain efficacy, but the use of organophosphates has become limited in some countries by environmental concerns and stricter registration procedures [15]. In 2010 to 2011, intense interventions using chemicals were triggered after a dengue epidemic in northern Brazil [16]. A rapid increase of pyrethroid resistance levels was detected and thought to be responsible for the inefficiency of deltamethrin treatments. The relationship between resistant genotypes and vector control also needs to be investigated more thoroughly. A survey conducted in Thailand showed that possession of 2 knockdown resistance (kdr) mutations in *Ae*. *aegypti* enabled survival after backpack pyrethroid spraying outdoors but not indoors, indicating that suboptimal interventions may select for specific mechanisms [17].

## Concepts underlying the evolution and spread of insecticide resistance and IRM

**Fig 1:**
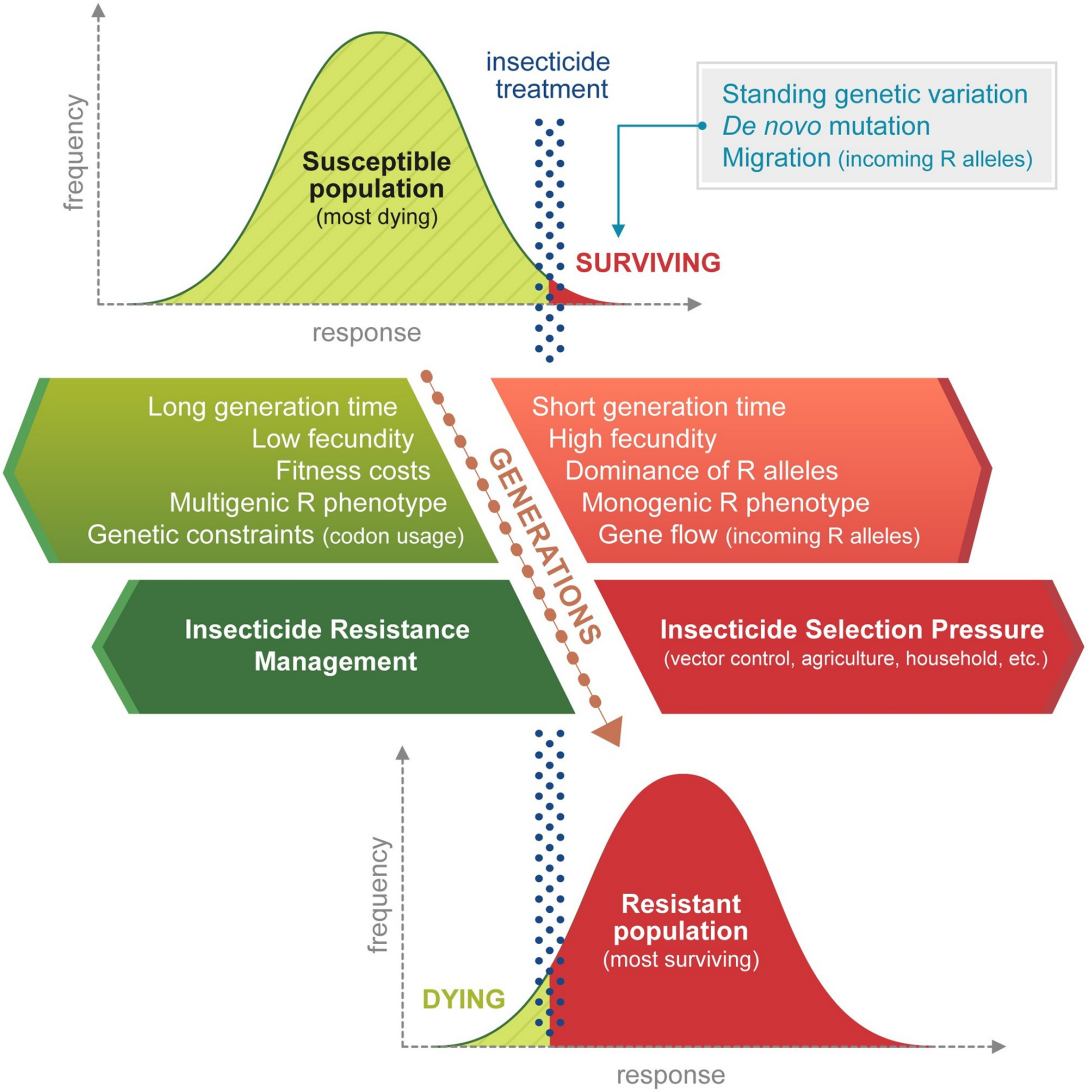
Factors affecting the selection of insecticide resistance in insect populations. The evolution of the population’s response to an operational dose of insecticide (red dotted line) across multiple generations of insecticide selection is shown. The proportion of individuals surviving insecticide exposure is shown in red. Factors favoring the selection of resistance are shown in red, while factors impairing selection of resistance including IRM are shown in green. IRM, insecticide resistance management.

The frequency of insecticide resistance in a population is an adaptive process driven by natural selection varying in space and time under the control of biological, genetic, and environmental factors (Fig 1). Although resistance can result from de novo mutations, such events are rare, and resistance in a population commonly arises from existing standing genetic variation (i.e., selection of rare resistant alleles) or from the arrival of individuals with resistance alleles through migration (or inadvertent transport by humans) [18, 19].

Besides insecticide selection pressure, biological and genetic parameters also drive the dynamics of resistance. Indeed, resistance will emerge faster in species showing high fecundity and short generation time. Furthermore, genetic constraints on codon usage may impair the selection of particular point mutations, as exemplified by the apparent lack of the G119S *ace-1* mutation in *Ae*. *albopictus* and a single detection in *Ae*. *aegypti* from India [20]. G119S conferring resistance to carbamates and organophosphates is common in *Anopheles* and *Culex* mosquitoes, for which only a single base substitution can change the wild type to a resistant allele [21]. The dominance and diversity of resistance alleles are also important. Resistance is more easily selected if the phenotype is caused by a few dominant alleles having a major effect rather than by multiple recessive alleles each having a minor effect. Finally, fitness costs associated with resistant alleles are of major importance as they represent a major evolutionary force driving the reversion of resistance in the absence of selection. Detection of fitness costs often involves comparing the dynamics of resistant alleles in natural populations in the absence or presence of selection pressure, although confounding factors may be difficult to eliminate [22]. Comparisons of life history traits among susceptible and resistant mosquito colonies under an environment free of insecticide in the laboratory are usually performed. Those data obtained under controlled conditions are easier to interpret, but those conditions are inevitably unrealistic and may involve laboratory colonies expressing an overly simplistic suite of resistance mechanisms, which may lead to overly optimistic predictions about resistance reversion [23, 24]. Of note, very few fitness cost studies have been conducted in *Aedes* mosquitoes; all have focused on *Ae*. *Aegypti*, and all have been performed under laboratory conditions (S1 Table). Resistance costs associated with kdr mutations that confer pyrethroid resistance have been identified, as have resistance costs associated with esterase-mediated organophosphate resistance and potential resistance to *Bacillus thuringiensis israelensis* (*Bti*) using a laboratory-selected strain. As with insecticide resistance itself, costs of resistance are unlikely to be static and may decrease over time after selection of additional resistant alleles or through compensatory pleiotropic effects [25–27]. Because the efficacy of IRM strategies to reverse resistance is partly dependent on the level of fitness costs, there is a clear need to characterize fitness costs associated with resistance to different insecticides in both *Ae*. *aegypti* and *Ae*. *albopictus* under realistic conditions and take into consideration other factors, such as susceptibility to predators and effects on vector competence [28, 29].

Although several factors can affect the dynamics of insecticide resistance in mosquitoes, the success of IRM once insecticide resistance has been detected relies almost entirely on the reduction of the insecticide selection pressure. However, insecticide resistance selection is not only due to vector control activities, as mosquitoes may also be exposed during their larval or adult stage to agricultural or household insecticides [30]. In addition, swapping insecticides with different modes of action would not affect the resistance phenotype if non–target-specific cross-resistance mechanisms (e.g., metabolic resistance) are important. In such cases, decreases in the frequency of resistance alleles will only occur if they are associated with significant fitness costs and if alternative insecticides are not impacted by the cross-resistance mechanisms.

## Insecticide resistance tracking

### Biological assays

Biological assays are commonly the first-line tests because they can reveal either the prevalence or level of phenotypic resistance. They are essential in the choice of control strategy and which alternative insecticides to use.

The principle underlying biological assays for resistance detection is to measure the response of batches of live insects after their exposure to an insecticide. Two main approaches allow resistance measurement (Table 1). First, mosquitoes are exposed to a fixed dose of insecticide for a given time (diagnostic dosage or concentration) that is expected to kill all susceptible individuals. The surviving ones after insecticide exposure are then considered resistant. This method is well adapted for routine monitoring but is not sensitive and may not provide a good measure of the intensity of resistance of an insect population. Moreover, few diagnostic doses are currently available, especially for *Aedes* spp. [31], encouraging the use of non–*Aedes*-specific or "homemade" diagnostic doses, which undermine the interpretation of results and comparisons between studies. Moreover, diagnostic doses are lacking for new molecules (e.g., clothianidin, chlorfenapyr, etc.) having increasing public health value.

**Table 1:** Advantages and disadvantages of methods for detection and monitoring of resistance in populations of insects (modified from [32]).

Methods	Advantages	Disadvantages	References
**Biological assays**			
*Diagnostic concentrations*	Standardized Simple and rapid to perform Detect resistance phenotype	Lack of sensitivity No information on level or type of resistance Few diagnostic doses available for *Aedes* spp. Require live mosquitoes Require universal quality insecticides	[31, 33]
*Dose-response assays*	Measure resistance levels	Require large number of live mosquitoes Require a susceptible reference colony	[34, 35]
*Assays using synergists*	Information on the potential mechanisms responsible for resistance	Lack of sensitivity and specificity Require large number of live mosquitoes	[33]
**Biochemical assays measuring enzyme activities**	Information on mechanisms responsible for resistance Several mechanisms tested on a single individual	Require a cold chain Not available for all resistance mechanisms Lack of sensitivity/specificity	[36]
**Molecular assays to detect resistant alleles**	Very sensitive Several mechanisms tested on single individuals Detect recessive alleles and provide an “early warning” of future resistance	Require specialized and costly equipment Only available for a limited number of resistance mechanisms Are not always easily linked to resistance levels	[37–40]

The second approach measures the response of insects that are exposed to a range of doses of insecticide, either by varying concentration or exposure time. The resulting dose-response analysis provides outcomes like the concentrations (or exposure times) that kill 50% or 90% of specimens. This allows the calculation of resistance ratios by comparison with the corresponding values from a susceptible strain [34, 35]. Dose-response assays better estimate resistance levels, but they require using a large number of mosquitoes and the use of a standard susceptible colony when available.

Bioassays should use technical-grade insecticides to avoid any effect caused by other compounds in the formulation. Batches of insects need to be as homogeneous as possible in terms of larval instar or physiological status and age for adults. For field mosquitoes, sampling effort should be sufficient to ensure that genotypic variability of insects is representative of the general population and that testing groups are not composed of highly related individuals. Several WHO guidelines were developed for monitoring insecticide resistance in mosquito populations [33, 41]

However, bioassays lack sensitivity to detect changes in susceptibility and often detect resistance only when the frequency of resistant alleles is already high, especially if resistance is recessive (e.g., kdr mutations). Unless associated with synergists, bioassays do not provide information on resistance mechanisms. Synergists are noninsecticidal molecules that block the activity of enzymes potentially involved in resistance. If these enzymes are involved in resistance, the insecticide is expected to recover its toxicity when the appropriate synergist is used. Synergists are used for metabolic resistance caused by esterases, monooxygenases, or glutathione-S-transferases [33], but their specificity to enzyme families is uncertain [42].

### Biochemical assays

Biochemical assays measure the activity or quantify the amount of detoxification enzymes, such as esterases, monooxygenases, and glutathione-S transferases in wild populations compared with a reference strain [36, 43]. They can also be used to characterize insensitive acetylcholinesterase by measuring the reduction of inhibition in the presence of organophosphates or carbamates. In these tests, a specific substrate is applied to enzyme extracts, and the products of substrate metabolism are quantified by a colorimetric reaction using a spectrophotometer or a spectrofluorometer. These assays require a cold chain to avoid loss of enzyme activities. Their specificity and sensitivity are flawed because only a few genes from a given enzyme family may be involved in resistance and the expression level of the whole enzyme family is not necessarily affected by the expression of enzymes causing resistance.

### Molecular assays

High-throughput molecular diagnostic tools associated with biological assays can provide key data for identifying the causes of resistance in order to implement adequate resistance management strategies [44]. In theory, detecting resistance alleles before operational resistance can be diagnosed by bioassays, or intervention failure can facilitate the management of resistance by indicating the need for a change of vector control tool before resistance alleles reach fixation. Molecular tools may help in choosing the best alternative insecticide, knowing the cross-resistance patterns associated with some resistance alleles that can metabolize insecticides from different unrelated families [45, 46].

Robust diagnostic tools have been developed for detecting kdr mutations associated with pyrethroid and DDT resistance in dengue vectors, mostly in *Ae*. *aegypti* [5]. These PCR-based assays can be performed on single mosquitoes, allowing the estimation of allele frequencies in multiple populations. They are now sometimes integrated into resistance monitoring programs. For instance, the findings of a nationwide distribution of kdr alleles in Brazil prompted the National Dengue Control Program to replace the pyrethroid adulticides in the whole country, even where resistance had not yet been confirmed by bioassays [47, 48]. Unfortunately, the alleles responsible for other mechanisms, such as metabolic resistance, are rarely investigated, which probably leads to frequent underestimation of their importance in natural populations. The current failure in the development of diagnostic assays for such resistance mechanisms has been mainly due to the lack of validated DNA markers. Fortunately, this problem is being addressed by the recent increase in high-throughput sequencing approaches, which promises to deliver new molecular assays for specific resistance mechanisms in *Aedes* mosquitoes [49].

## A decision framework toward implementation of IRM

**Fig 2:**
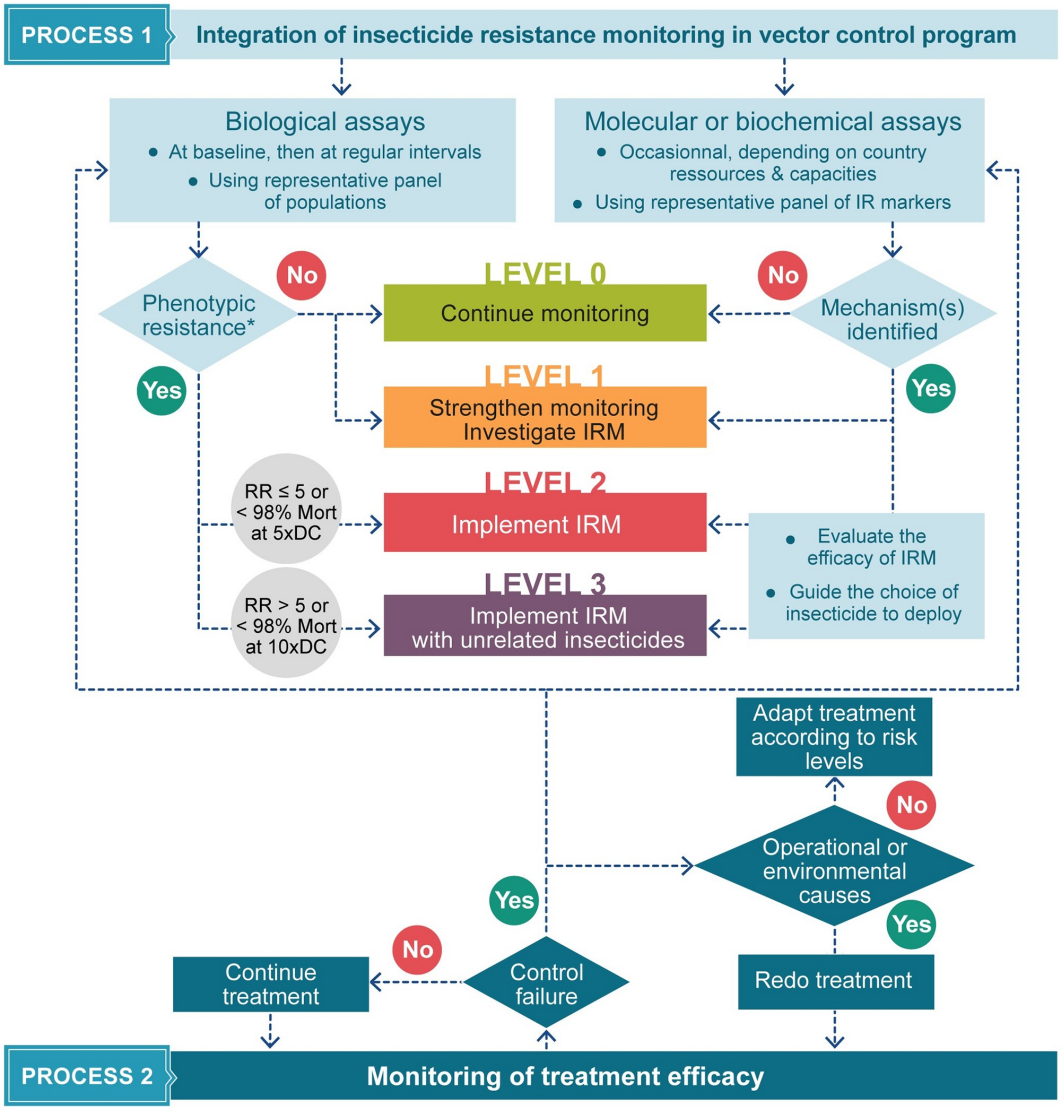
Flow chart to support decision-making of IRM strategy during implementation of a vector control program. The first process deals with resistance monitoring within the target insect population. The second process is the monitoring of treatment efficacy that should be run in parallel with process 1. It aims to detect any control failure and whether it is caused by resistance or other external factors. Risk levels are defined according to the results of resistance monitoring and should trigger graduated and appropriate response: (i) level 0 indicates a population fully susceptible to the insecticide, (ii) level 1 designates a population whose susceptibility is maintained but some of whose individuals harbor resistant alleles, (iii) level 2 corresponds to a moderate resistance (e.g., RR below 5 or below 98% mortality using 5 times the WHO DC), (iv) level 3 corresponds to populations clearly resistant to a given insecticide and that require immediate IRM strategy (e.g., RR above 5 or below 98% mortality using 10 times the WHO DC). According to the current knowledge gap, molecular or biochemical assays cannot be straightforwardly used to define IRM levels (except from level 0 to level 1), and basically, these levels are defined using the bioassays. The resistance thresholds for levels 2 and 3 are only indicative and fixed by analogy to the last WHO procedures [33]. They should be refined according to operational-based evidences. For levels 2 and 3, the characterization of resistance mechanisms is requested to guide a decision on alternative insecticides and to follow the impact of IRM on the frequency of resistance alleles. DC, diagnostic concentration; IR, insecticide resistance; IRM, insecticide resistance management; Mort, mortality; RR, resistance ratio.

Before implementation of any vector control program, it is of primary importance to determine the susceptibility of target populations to the insecticide(s) that will be used. Sentinel sites that are representative of the area to be treated should be identified and regularly monitored during operations. A flow chart to support decision-making for IRM strategy during implementation of vector control program is given in Fig 2.

The main objective of process 1 is the implementation of resistance monitoring on a regular basis through biological assays. This action should be taken by vector control agencies to ensure a fast and appropriate response. In practice, control agencies do not always have the facilities or the human and technical resources to conduct bioassays, underscoring the need for strengthening their capacities in routine entomological surveillance and resistance monitoring. Long-term monitoring allows detection of possible decreases in susceptibility in field populations, but the relationship between bioassays results and operational failure still needs to be assessed. During this process, it is important to search for mechanisms that can be involved in resistance to the products employed. These techniques require specific skills and equipment, and core laboratories in research institutions usually carry them out. It is therefore important to build communication and collaboration among academic research and control agencies to increase knowledge transfer and the contributions from basic research to field operations. The objective is to detect emergence of resistance long before operational failures occur. Detecting an increase in resistant alleles in a population should give sufficient time for the operating manager to modify the vector control strategies. However, the timescale over which such a frequency change might forewarn of operational failure remains unclear, and more work is required to investigate the link between resistance mechanisms and high-level resistance that could trigger an operational failure.

Process 2 aims to detect control failures in the field and to identify their possible causes. Control failure is defined here as a significant reduction of efficacy of an insecticide formulation used at recommended dose/concentration but not reaching an expected control level [50]. The choice of indicators to monitor efficacy should depend on the nature of treatments, e.g., larval abundance or time for the apparition of newly emerged larvae for larvicides, females’ density using various trapping methods for adulticides. Many reasons other than insecticide resistance can be suspected when a control operation is not successful. It is important to consider all possibilities before inferring that the failure is due to insecticide resistance, including the quality of the insecticide used, the accuracy of insecticide dosage and coverage, or treatment frequency. Additionally, external factors, such as environmental conditions that can affect the quality of application (e.g., wind, rain, temperature), need to be considered. If all operational or environmental factors can be ruled out and if insecticide resistance is confirmed in target populations, then adaptive measures for control strategies should be implemented in a graduated way according to the risk level. The criteria used to define a risk level based on the thresholds for mortality rates or resistance ratios quantified by bioassays should be refined according to operational evidence of their corresponding impact in the field. At level 1, investigations may be done by testing the susceptibility of field populations to potential alternative insecticides. At levels 2 and 3, the resistance markers allow generating data to improve the correlation between genotypes and operational consequences or to follow the impact of an IRM strategy on gene evolution and then on a middle term to support decision-making.

## IRM strategies: Past and current experiences

Several strategies are available for managing resistance. They include approaches that reduce the fitness of resistant individuals or that reduce the overall selection pressure exerted on insect populations [51]. Although many strategies have been developed, especially in agricultural settings, the main strategies considered for public health are rotations, mosaics, and mixtures of insecticides, among which there is no cross-resistance, i.e., they have different mode of actions and are not targeted by the same metabolic/cuticular mechanisms (Box 1).

**Box 1. IRM strategies**

**Rotation**

This strategy employs temporal alternation of insecticides with different modes of action and is based on the assumption that resistance diminishes insect fitness in the absence of insecticide. Emerging resistance is expected to require time to establish in a population, and the frequency of resistant individuals should drop after application of an alternative insecticide. The rotation frequency is designed to provide insufficient time for a significant increase in alternate resistance mechanisms in the population. Therefore, rotations must be done at a relatively high frequency, with switch rate determined by the residual efficacy of treatments and the generation time of target insects [52, 53]. In the case of public health vectors, a 1-year rotation is usually adopted for indoor residual spraying (IRS), but it should be shorter for space sprays or larvicidal treatments.

**Mosaic**

A mosaic strategy involves the spatial alternation of 2 or more insecticides with different modes of action. The insect population from one treated area is exposed to insecticide A, while the population from an adjacent area is exposed to insecticide B. This strategy works better when movement of insects across differentially treated areas is high enough to "dilute" the frequency of resistance alleles and when a strong selection against resistance occurs in areas treated with alternative insecticide [54]. A mosaic strategy can be used in different houses within one locality, different districts within one city, or at the scale of different villages depending on the flight range of the targeted species. However, Aedes commonly have very short flight ranges [55], which may limit the applicability of a broad-scale approach.

**Mixture of insecticides**

A mixture is the concurrent use of two or more insecticides with different modes of action. The use of mixtures is based on the hypothesis that if the probability of developing resistance or the frequency of resistance alleles is low then individuals with multiple resistance mechanisms will be very rare. To be effective it is also important that there is no cross-resistance between insecticides, that insecticides have the same residual efficacy–so as to maintain the ratio between active ingredients, and that resistance is functionally recessive (i.e. only homozygous resistant for both mechanisms can survive the mixture [56, 57]. Mixtures are often used in agriculture to control resistant pests or different insect species, and there are unfortunately still very few mixed products currently approved for public health vectors.

### IRM in public health vectors

It is clear that IRM must be part of vector control strategies. However, to date, only a few strategic plans have enforced an integrated vector management (IVM) that includes insecticide resistance monitoring, alternative methods, and IRM against *Aedes* species [58, 59].

To date, attempts to manage insecticide resistance have largely failed in *Aedes* populations, probably because resistance levels were already too high and well established when IRM was implemented [3]. The current standard response of control programs to *Aedes* resistance is to change insecticide once treatments become less effective or even ineffective. This “reactive alternation” is a pragmatic approach but should not be considered as an IRM strategy. All comparisons with mosaic, rotation, or mixture based on theoretical models or empirical studies demonstrate that “reactive alternation” underperforms the other IRM approaches in slowing down resistance evolution because alternation is implemented too late, when resistance alleles are already at high frequency [60]. In Singapore, permethrin was replaced by pirimiphos-methyl for thermal fogging after reduced efficacy was observed [61], yet after 9 years of interruption, high permethrin resistance persisted in field populations. Persistence of high levels of pyrethroid resistance was also observed 7 to 10 years after they were replaced by organophosphates in the state of Sao Paulo (Brazil) [62]. As mentioned before, however, maintenance of resistance can also be due to the exposure of vector populations to household and agricultural insecticides or other xenobiotics (pollutants, other pesticides, etc.) [63–65].

On the bright side, the Onchocerciasis Control Programme in West Africa is probably the most emblematic case of successful IRM in public health. After the first detections of temephos resistance in *Simulium damnosum*, a rotation scheme of insecticides with alternative modes of action was implemented for larval treatments. The rotation strategy drove the reversion of organophosphate resistance and preserved *S*. *damnosum* insecticide susceptibility for more than 20 years until the program was stopped [66].

For malaria, a large-scale trial was conducted in Mexico to evaluate the impact of different IRM strategies on the resistance of *Anopheles albimamus* [67]. In this study, a 3-compound annual rotation, a 2-compound mosaic, and a long-term treatment with a single insecticide were compared. An increase of resistance was observed in all villages, but it demonstrated that mosaic or rotation selected for low resistance levels and remained stable compared with villages with single treatment where resistance increased rapidly and to levels significantly greater [68]. Control failures associated with pyrethroid resistance in malaria vectors have been observed for IRSs using pyrethroids. Switching to DDT against *An*. *funestus* populations without kdr mutation but specific metabolic mechanisms of pyrethroid resistance in South Africa [69] or to bendiocarb against *An*. *gambiae* in Equatorial Guinea restored the control of resistant populations [70].

Specific IRM strategies targeting the *Aedes* genus may be more likely to succeed if they target immature stages rather than imago. Firstly, larvae have no possibility to escape the treatment, reducing the possibility for behavioral resistance contrary to adults. Secondly, several larvicides covering 4 chemical classes (organophosphates, juvenile hormone mimics, chitin synthesis inhibitors, and spinosyns), plus a biological agent, *Bti*, are available for larval control, all of which could be rotated easily, in contrast to space sprays that rely solely on 2 chemical classes (pyrethroids and organophosphates). *Bti*, due to its unique mode of action as a mixture of toxins, has a high potential for *Aedes* control and resistance management. After more than 30 years of use, no resistance has been reported so far [5]. The product is cheap, safe, registered in many countries, and can then be widely deployed in regions where mosquitoes have become resistant to chemical insecticides. In areas where IRS and targeted IRS can be massively deployed for dengue control, pyrethroids should not be used as a single treatment but as part of a coordinated rotation using nonpyrethroid insecticides [71]. The use of noninsecticidal tools is an efficient way to reduce the selection pressure on vector populations [72], and it has the advantage to be applied within an IVM strategy, which aims to improve vector control efficacy while optimizing the use of available resources [58].

### Lessons from IRM in agriculture

There are some important differences between insecticide-based vector control in public health and pest control in agriculture. In contrast to crop protection, for which a relatively large number of active ingredients and types of insecticides are available, only a very limited number are registered for use in public health due primarily to the greater potential for human exposure (Table 2). Common insecticide profiles are also different and impose very different selection pressures. Critically, formulations used in agriculture commonly have low residual activity, whereas vector control insecticides used in certain applications (e.g., IRS, insecticide-treated material, long-lasting larvicides as insect growth regulators) should have long enough residual effects to cover the primary transmission season. Where this is not the case, repeated reapplication is required increasing costs and logistical demands.

**Table 2:** IRM practices in public health and agriculture.

Public Health (Vector Control)	Agriculture (Crop Protection)
***Insecticides***	
Very few AIs/modes of action	Many different AIs/modes of action
High residual activity formulations (high selection pressure)	Low residual activity formulations (adjustable selection pressure)
***Resistance diagnosis/monitoring***	
WHO/CDC bioassays: no direct link with impact of resistance on efficiency	IRAC bioassays: direct association with field application rates and control failure
Advanced biochemical/molecular diagnostics	Simple molecular diagnostics
***Risk assessment***	
Researchers, WHO	Industries–regulatory bodies: test methods, in advance monitoring, resistance management guidance
***IRM***	
Delayed implementation of IRM: (Researchers, WHO, national control programs).	Early implementation of IRM: Plans in place (guidelines) before use (industry, regulatory bodies, researchers)
Rotations of limited number of AIs Mixtures rarely used Mosaics rarely used	Rotations of large number of AIs Mixtures Mosaics Restricted number of applications per period, per crop, per pest, per region Refugee crops
General guidelines, but limited evidence-based local/regional guidelines	No common strategies globally, but several local/regional robust guidelines
***IVM/IPM***	
Environmental management, biological control, larvicides, alternative tools; Limited validated/WHO recommended options	Resistant varieties, biological control, GMOs, alternative products (green chemistry), etc.
***Communication of IR data***	
Researchers, stakeholders, WHO, industry (limited)	Industry, end users, researchers
Publications and reports Vector control decision support system Disease data management system IR Mapper	Publications and reports Global Pesticide Resistance Database (Michigan), IRAC

**Abbreviations:** AI, active ingredient; CDC, US Centers for Disease Control and Prevention; GMO, genetically modified organism; IPM, integrated pest management; IR, insecticide resistance; IRAC, Insecticide Resistance Action Committee; IRM, insecticide resistance management; IVM, integrated vector management; WHO, World Health Organization

The WHO definition of resistance was based on the ability of insects to tolerate doses of toxicants that are lethal to the majority of individuals in a normal population of the same species [73]. Then, bioassays in public health were developed to detect a significant decrease of susceptibility within field populations compared with a susceptible population or colony. They use technical-grade insecticides and diagnostic doses/concentrations that do not reflect the operational ones. In crop protection, the bioassays are more intended to identify insecticide resistance having the potential of control failure in the field [50]. These tests are established by the Insecticide Resistance Action Committee (IRAC), and they are usually based on formulated products at doses/concentrations recommended for field applications. The insecticide industry in crop protection has been urged to include a comprehensive risk assessment in their registration files, describing robust testing methods and demonstrating efficiency against large numbers of field populations. Therefore, IRM often starts early before or just after active ingredients are first used, and several stakeholders are involved. In contrast, vector control IRM is typically a response to reports of possible insecticide failures, often after many years of insecticide use. The most striking example is the time for development of a global plan for IRM [74] in malaria vectors approximately 20 years after the first report of pyrethroid resistance in *Anopheles* mosquitoes in West Africa [75].

Similar tactics are implemented in both crop protection and vector control IRM. However, the evidence that common agricultural IRM strategies such as rotations and mixtures work in vector control settings remains very limited [74]. It is assumed that these are the correct strategies to manage resistance in vector control as well, but more operational research is needed on this subject.

However, the value of insecticide in a framework of integrated pest management (IPM) or IVM is indisputable. The concept of IVM benefited from developments of IPM in agriculture and is defined as a rational decision-making process to optimize the use of resources to make vector control more efficient, cost effective, ecologically sound, and sustainable [58]. In the management of resistance, the importance of alternative tools in crop protection (such as resistant plant varieties, biological control, etc.) and equivalent vector control alternatives (such as environmental management, traps, repellents, genetic methods, sterile insect technique [SIT], or *Wolbachia*-based insect incompatible technique [IIT]) is recognized [72]. However, a major contrast to crop protection, in which demonstration of efficacy is relatively straightforward, is the requirement for epidemiological impact in vector control. Indeed, the epidemiological impact and the scope of application of new vector control tools—such as attractive toxic sugar baits, spatial repellents, SIT, *Wolbachia*-based IIT, or genetically modified mosquitoes—has not yet been well established, precluding current recommendations by WHO.

Finally, appropriate and effective means to communicate on insecticide resistance and other entomological data are available both in vector control and crop protection. However, training and education on how to interpret these data to address specific operational questions and make decisions are limited, particularly in the vector control area.

In conclusion, some lessons from the IRM in crop protection should be considered in mosquito vector control IRM: (i) earlier development and implementation of IRM, (ii) involvement of more stakeholders, (iii) more active ingredients required, (iv) earlier use of alternative control tools in the framework of integrated management, (v) improved diagnostics providing information about the impact of resistance on control, (vi) sustainable communication of IRM guidance and data, and (vii) advanced training of managers to interpret and use insecticide resistance data for timely decision-making.

## Toward improved IRM for sustaining the control of arbovirus mosquito vectors

The implementation of IRM in public health is particularly challenging due to the difficulty of convincing the public authorities and stakeholders of its benefits. This is mostly explained by the lack of evidence to link resistance-associated control failures to increased dengue, Zika, or chikungunya transmission. Because vector control aims to reduce or prevent infection or disease to humans, any tools or strategies intended for IRM should have proven an epidemiological impact. However, even for most control methods that are currently in widespread use, there are little data demonstrating their efficacy on incidence of infection and disease [76, 77]. The public health value of an intervention against vectors is evaluated under large-scale trials, preferably using a randomized controlled design. These trials are complex to implement, expensive, time-consuming, and usually not considered high priority by funding agencies. The limited evidence-based information to support any IRM strategy in public health (e.g., mixture, rotation, noninsecticidal tools, etc.) complicates the prioritization of interventions.

Lastly, there is no harmonized regulatory or legislative framework for the implementation of vector-related activities at the country and/or regional level. This raises the question not only for implementing IRM but also for deploying and evaluating new vector control tools/strategies, e.g., *Wolbachia* based, genetically modified mosquitoes, etc.

Despite that, we have to advocate for the introduction of IRM policies into vector control programs by favoring a proactive approach over a response mode. For example, it has been demonstrated that the preset rotation of unrelated insecticides performed better than changing insecticides over time in response to resistance [60, 66]. Preserving insecticide susceptibility is extremely valuable over the long term and should be recognized by WHO and stakeholders as a public good and protected accordingly [78]. Vector control is more driven by short-term economics (favoring tools with minimum costs for maximum coverage) than by the middle- or long-term value for sustaining insecticide susceptibility. Hence, advocacy is needed to make the case by promoting education on the benefit of resistance management strategies to preserve the susceptibility of vector populations. The case should be made for benefits of intersectoral collaboration, community involvement, cost-effectiveness of vector control, potential cost savings (reducing insecticide use, pollution, side effects), and fostering innovation. There is a need to develop educational material on IRM as well as technical guidance for countries to incorporate good management practices in a national strategic framework for vector control. The Worldwide Insecticide resistance Network (WIN) initiative aims to gain support for and reduce barriers to those initiatives [79] with the scope to diminish the burden of *Aedes*-borne diseases by 2030.

The vector control strategies at the country level should be based on a resistance monitoring system and the planning and implementation of IRM. The 2 sets of activities should interact constantly and should be updated regularly, as change in the resistance situation in the field requires adaptation of the vector control plan and inversely, operational change needs adaptation of the resistance monitoring system.

Research and innovative tools are needed and should be focused on the 3 key elements expected to significantly impact the efficacy and sustainability of IRM:

- Control tools: In the short term, an IRM strategy can be developed quickly using available public health insecticides. Although mixture or rotations have rarely been used, some mixtures of existing insecticides have been found very efficient against multiresistant larvae of Caribbean *Ae*. *aegypti* [80] or against pyrethroid-resistant adult mosquitoes [81, 82]. However, in the midterm, it will be necessary to integrate novel insecticides with new modes of action. For example, the Innovative Vector Control Consortium, though primarily focused on the control of malaria vectors, has—with industrial partners—developed insecticides that should become available for use against arbovirus vectors [6]. Additionally, the use of alternative, innovative methods—including repellents, traps, genetically modified mosquitoes, SIT, or *Wolbachia*-based methods—could become part of an integrated vector control strategy [72] once efficacy and safety has been fully evaluated, because they contribute to the reduction of insecticide use and consequently to the selection pressure on vector populations.
- Monitoring tools: In the short term, bioassays and methodologies to monitor insecticide resistance in *Aedes* need to be developed so we can obtain reliable and comparable datasets. CDC bottle tests and WHO tube tests are often used interchangeably for resistance monitoring [83]. Those assays may be efficient for detecting resistance, but they are not directly comparable [84]. Furthermore, diagnostic concentrations need to be determined for *Aedes* spp. and for all insecticides recommended by WHO. In the midterm, molecular markers of resistance mechanisms should be systematically identified and their predictive value quantified to allow sensitive tracking of fluctuations in resistance following environmental changes and vector control operations. However, many alleles associated with metabolic/cuticular resistance have not yet been identified and are usually monitored through gene expression studies unsuitable for routine detection. Recent studies using next-generation sequencing identified promising DNA markers in dengue vectors [49, 85–87]. Such DNA-based diagnostic tools should be robust and easy to run, have high throughput, and be affordable in order to be readily implemented in resistance monitoring programs worldwide.
- Knowledge improvement of resistance significance: Until now, it has been near impossible to anticipate how resistance will impact the efficacy of vector control tools in the field and how a given IRM strategy will slow or reverse the evolution of resistance in targeted populations. A continuous effort connecting research institutes, control programs, industry, and WHO is required to conduct operational research that evaluates vector control strategies under field conditions and the genetics of resistance in broad terms (genotype–phenotype relationships, mosquito behavior, modeling). The outcomes of these studies will provide crucial information to optimize decision-making systems and the procedures to adjust IRM strategy according to field situations.

**Fig 3:**
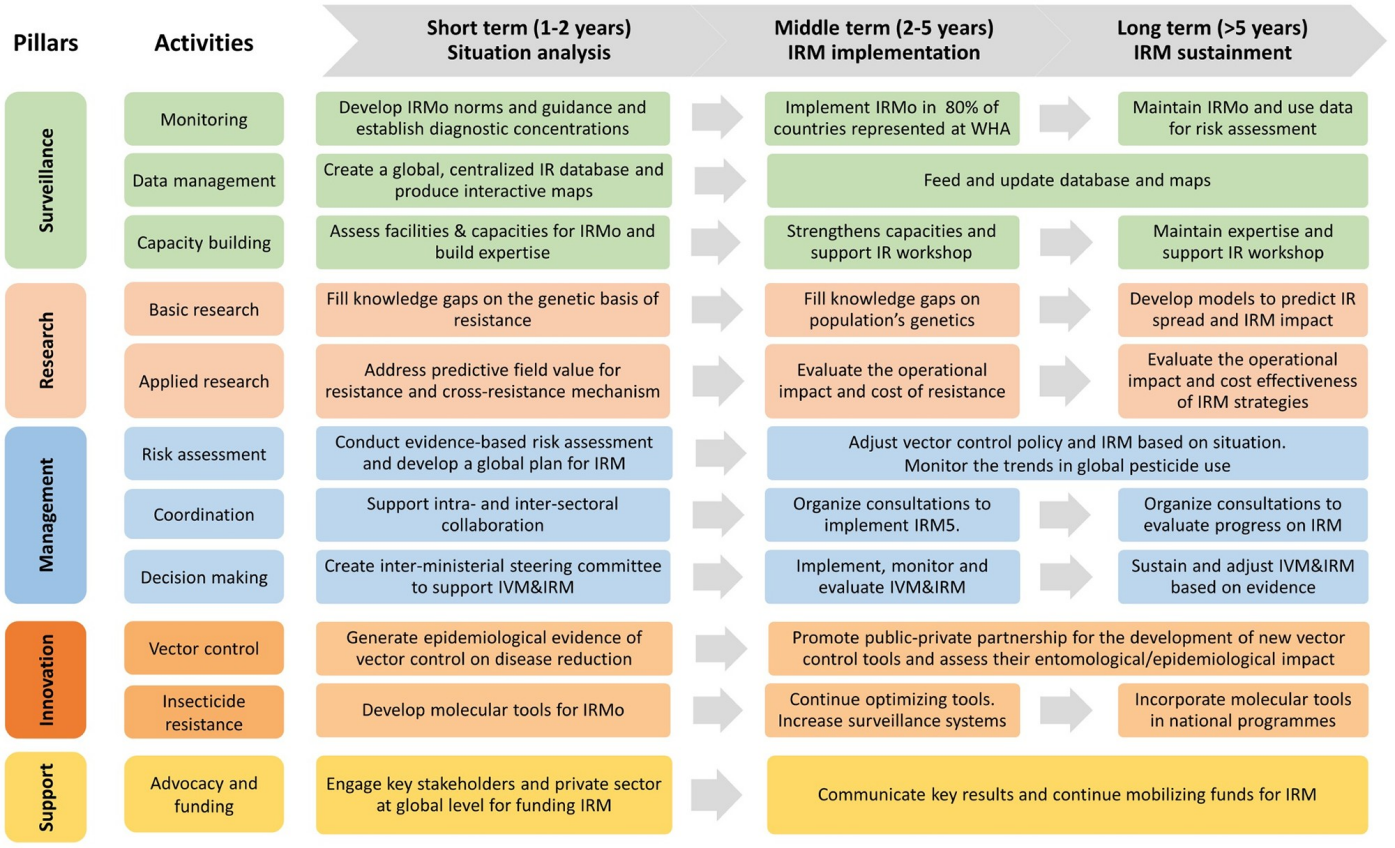
Overview of the key activities required to devise and implement IRM plan in *Aedes* spp. in the future. The global plan is based on 5 pillars including 1 to 3 key activities. Timelines of different key activities are proposed to serve as progress indicators for the different stakeholders (control programs, funding agencies, WHO, research institutes, etc.). IR, insecticide resistance; IRM, insecticide resistance management; IRMo, insecticide resistance monitoring; IVM, integrated vector management.

In recognition of the tremendous liability that insecticide resistance in *Anopheles* spp. represents for malaria control programs, a Global Plan for Insecticide Resistance Management (GPIRM) was prepared and published by WHO [74]. The plan proposes a collective strategy for the malaria community to overcome resistance challenge and to maintain the efficacy of vector control tools [88]. Even if the implementation of GPIRM at national level has been challenging for many reasons, a similar plan for arbovirus vectors would be valuable for enabling efficient monitoring and management of *Aedes* resistance at a global scale. Here, we propose a framework for driving future development of a global plan for IRM in arbovirus vectors (Fig 3).

## Conclusion

Increases in insecticide resistance development in *Aedes* vectors as arbovirus epidemics proliferate underscore the urgency to create IRM programs to maintain or recover vector control efficacy. When control strategies using insecticides are implemented, they should be systematically associated with noninsecticidal tools and, when possible, replaced by alternative tools to reduce the selection pressure on *Aedes* populations and limit the evolution of resistance. Such advancement needs coordination, training, and development of new tools. The WIN supported by WHO has demonstrated its capacity to federate worldwide researchers, public health authorities, and stakeholders to work together and establish a global plan to improve *Aedes* vector control. We aim to provision, widen, and strengthen the WIN network in order to develop and implement *Aedes* IRM at a global scale.

**Key learning points**

- IRM aims to prevent the emergence of, slow down the evolution of, or reverse insecticide resistance in populations by employing strategies compatible with efficient vector control but a limited effect on the environment.
- The impact of *Aedes* insecticide resistance on control failure needs to be further evaluated to better assess risk thresholds for decision-making.
- IRM success relies on the reduction of insecticide pressure sources, especially vector control, but also from agricultural and domestic use.
- Regular insecticide resistance monitoring by biological and molecular assays as well as evaluation for vector control failure are key processes for IRM.
- Strategies for IRM include insecticide rotation, mosaic, and mixture/combination as well as the use of nonchemical alternatives in the frame of IVM, but there have been few tests of different strategies for efficacy on *Aedes* populations and mosquitoes generally.
- There is a need to support research for the development of surveillance and control tools to improve IRM strategies.
- Widespread and increasing insecticide resistance and outbreaks of *Aedes*-driven arboviruses make the development of a global plan for IRM in *Aedes* vectors an urgent need.

**Top five papers**

- 1. Moyes CL, Vontas J, Martins AJ, Ng LC, Koou SY, Dusfour I, et al. Contemporary status of insecticide resistance in the major *Aedes* vectors of arboviruses infecting humans. PLoS Negl Trop Dis. 2017;11(7):e0005625. doi: 10.1371/journal.pntd.0005625. PMID: 28727779.
- 2. Sternberg ED, Thomas MB. Insights from agriculture for the management of insecticide resistance in disease vectors. Evol Appl. 2018;11(4):404–14. doi: 10.1111/eva.12501. PMID: 29636795
- 3. Roiz D, Wilson AL, Scott TW, Fonseca DM, Jourdain F, Muller P, et al. Integrated Aedes management for the control of Aedes-borne diseases. PLoS Negl Trop Dis. 2018;12(12):e0006845. doi: 10.1371/journal.pntd.0006845. PMID: 30521524
- 4. Vontas J, Kioulos E, Pavlidi N, Morou E, della Torre A, Ranson H. Insecticide resistance in the major dengue vectors *Aedes albopictus* and *Aedes aegypti*. Pestic Biochem Physiol. 2012;104(2):126–31. doi: 10.1016/j.pestbp.2012.05.008.
- 5. WHO. Global plan for insecticide resistance management in malaria vectors. http://www.who.int/malaria/publications/atoz/gpirm/en/, editor. Geneva, Switzerland: Global Malaria Programme—Vector Control Unit; 2012. 130 p.

## Supporting information

S1 Table Summary of studies investigating costs of resistance in *Ae*. *aegypti*. (DOCX)
