# Supplementary material for: Management of insecticide resistance in the major Aedes vectors of arboviruses: Advances and challenges
Source: PLoS Negl Trop Dis. 2019 Oct 10;13(10):e0007615. doi: 10.1371/journal.pntd.0007615 (PMC6786541; doi:10.1371/journal.pntd.0007615)
Supplement: S1 Table — (DOCX) [file pntd.0007615.s001.docx]

**S1 Table. Summary of studies investigating costs of resistance in *Aedes* (all *Ae. aegypti*)**

| Source | selection regime | comparison | resistance | mechanism(s) | LdR | AdS | Lon | Siz | Fed | Fec | Ins | Ref |
| --- | --- | --- | --- | --- | --- | --- | --- | --- | --- | --- | --- | --- |
| Brazil (field lines) | diflubenzuron: 6-7 g | selected/unselected | ≈4x | n/a | n/a | n/a | **↓** | n/a | **↓** | **↓** | **↓** | [1] |
| Rockefeller strain | 1016I+1534C crossed into Rockefeller | Rock-kdr/Rockefeller | ≈100x | *kdr* 1016I+1534C | **↓** | = | = | n/a | n/a | **↓** | n/a | [2] |
| Taiwan strain (Per-R) | permethrin selection *withdrawn* for 15 g | resistant/resistance reversed | 5x | *kdr* V1016I+D1763Y | n/a | n/a | n/a | n/a | n/a | n/a | n/a | [3] |
| Brazil (Rec-R strain) | temephos selection *withdrawn* for 21 g | resistant/resistance reversed | 40x | esterases, GSTs | **↓** | **↓** | **↓** | n/a | n/a | **↓** | n/a | [4] |
| Brazil (3 field lines) | deltamethrin: 9 g | selected/unselected | 6x | n/a | **↓** | n/a | **↓** | n/a | **↓** | **↓** | n/a | [5] |
| Thai strain (PMD-R) | permethrin: 10 years | selected/unselected | n/a | *kdr* F1534C | = | = | = | **↓** | n/a | **↑** | = | [6] |
| Colombia (field line) | lambda-cyhalothrin for 9-10 g | selected/unselected | 8-22x | n/a | = | = | **↓** | **↓** | n/a | **↓** | n/a | [7] |
| Bora Bora strain | 3 *Bti* toxins: up to 22 g | selected/unselected | 6-35x | n/a | **↓** | = | = | = | n/a | **↓*** | = | [8] |

g, generations; *Bti, Bacillus thuringiensis israelensis*; n/a, not applicable or available;↓, significant reduction (evidence for 'cost'); ↑, significant increase; =, no significant difference; ↓*, significant reduction and reduced dried egg viability; LdR, larval development rate; AdS, survival to adult; Lon, longevity; Siz, adult size; Fed, blood-feeding; Fec, fecundity; Ins, insemination rate

1. Belinato TA, Valle D. The Impact of Selection with Diflubenzuron, a Chitin Synthesis Inhibitor, on the Fitness of Two Brazilian Aedes aegypti Field Populations. PLoS ONE. 2015;10(6):e0130719. doi: 10.1371/journal.pone.0130719. PMID: 26107715

2. Brito LP, Linss JG, Lima-Camara TN, Belinato TA, Peixoto AA, Lima JB, et al. Assessing the effects of Aedes aegypti kdr mutations on pyrethroid resistance and its fitness cost. PLoS ONE. 2013;8(4):e60878. doi: 10.1371/journal.pone.0060878. PMID: 23593337

3. Chang C, Huang X-Y, Chang P-C, Wu H-H, Dai S-M. Inheritance and stability of sodium channel mutations associated with permethrin knockdown resistance in Aedes aegypti. Pestic Biochem Physiol. 2012;104(2):136-42. doi: <http://dx.doi.org/10.1016/j.pestbp.2012.06.003>.

4. Diniz DF, de Melo-Santos MA, Santos EM, Beserra EB, Helvecio E, de Carvalho-Leandro D, et al. Fitness cost in field and laboratory Aedes aegypti populations associated with resistance to the insecticide temephos. Parasit Vectors. 2015;8:662. doi: 10.1186/s13071-015-1276-5. PMID: 26715037

5. Martins AJ, Ribeiro CD, Bellinato DF, Peixoto AA, Valle D, Lima JB. Effect of insecticide resistance on development, longevity and reproduction of field or laboratory selected Aedes aegypti populations. PLoS ONE. 2012;7(3):e31889. doi: 10.1371/journal.pone.0031889. PMID: 22431967

6. Plernsub S, Stenhouse SA, Tippawangkosol P, Lumjuan N, Yanola J, Choochote W, et al. Relative developmental and reproductive fitness associated with F1534C homozygous knockdown resistant gene in Aedes aegypti from Thailand. Trop Biomed. 2013;30(4):621-30. PMID: 24522132.

7. Jaramillo ON, Fonseca-Gonzalez I, Chaverra-Rodriguez D. Geometric morphometrics of nine field isolates of Aedes aegypti with different resistance levels to lambda-cyhalothrin and relative fitness of one artificially selected for resistance. PLoS ONE. 2014;9(5):e96379. doi: 10.1371/journal.pone.0096379. PMID: 24801598

8. Paris M, David JP, Despres L. Fitness costs of resistance to Bti toxins in the dengue vector Aedes aegypti. Ecotoxicology. 2011;20(6):1184-94. doi: 10.1007/s10646-011-0663-8. PMID: 21461926
